# Concern over radiation exposure and psychological distress among rescue workers following the Great East Japan Earthquake

**DOI:** 10.1186/1471-2458-12-249

**Published:** 2012-05-15

**Authors:** Yutaka Matsuoka, Daisuke Nishi, Naoki Nakaya, Toshimasa Sone, Hiroko Noguchi, Kei Hamazaki, Tomohito Hamazaki, Yuichi Koido

**Affiliations:** 1 Department of Psychiatry, National Disaster Medical Center, 3256 Midoricho, Tachikawa, 190-0014, Japan; 2 Clinical Research Institute, National Disaster Medical Center, 3256 Midoricho, Tachikawa, 190-0014, Japan; 3 Translational Medical Center, National Center of Neurology and Psychiatry, 4-1-1 Ogawahigashi-cho, Kodaira, 187-8551, Japan; 4 National Institute of Mental Health, National Center of Neurology and Psychiatry, 4-1-1 Ogawahigashi-cho, Kodaira, 187-8553, Japan; 5 CREST, Japan Science and Technology Agency, 3256 Midoricho, Tachikawa, 190-0014, Japan; 6 Department of Nutrition and Dietetics, Faculty of Family and Consumer Sciences, Kamakura Womens University, 6-1-3 Ofuna, Kamakura, 247-8512, Japan; 7 Department of Rehabilitation, Faculty of Health Science, Tohoku Fukushi University, 1-8-1 Kunimi, Sendai, 981-8522, Japan; 8 Department of Public Health, Faculty of Medicine, University of Toyama, 2630 Sugitani, Toyama, 930-0194, Japan; 9 Department of Clinical Sciences, Institute of Natural Medicine, University of Toyama, 2630 Sugitani, Toyama, 930-0194, Japan; 10 Japan Disaster Medical Assistance Team, 3256 Midoricho, Tachikawa, 190-0014, Japan

## Abstract

**Background:**

On March 11, 2011, the Great East Japan Earthquake and tsunami that followed caused severe damage along Japans northeastern coastline and to the Fukushima Daiichi nuclear power plant. To date, there are few reports specifically examining psychological distress in rescue workers in Japan. Moreover, it is unclear to what extent concern over radiation exposure has caused psychological distress to such workers deployed in the disaster area.

**Methods:**

One month after the disaster, 424 of 1816 (24%) disaster medical assistance team workers deployed to the disaster area were assessed. Concern over radiation exposure was evaluated by a single self-reported question. General psychological distress was assessed with the Kessler 6 scale (K6), depressive symptoms with the Center for Epidemiologic Studies Depression Scale (CES-D), fear and sense of helplessness with the Peritraumatic Distress Inventory (PDI), and posttraumatic stress symptoms with the Impact of Event Scale-Revised (IES-R).

**Results:**

Radiation exposure was a concern for 39 (9.2%) respondents. Concern over radiation exposure was significantly associated with higher scores on the K6, CES-D, PDI, and IES-R. After controlling for age, occupation, disaster operation experience, duration of time spent watching earthquake news, and past history of psychiatric illness, these associations remained significant in men, but did not remain significant in women for the CES-D and PDI scores.

**Conclusion:**

The findings suggest that concern over radiation exposure was strongly associated with psychological distress. Reliable, accurate information on radiation exposure might reduce deployment-related distress in disaster rescue workers.

## Background

On March 11, 2011, the magnitude 9.0 Great East Japan Earthquake and tsunami that followed devastated the northeastern coast of Japan and damaged the Fukushima Daiichi nuclear power plant. Members of the Disaster Medical Assistance Team (DMAT) service, established by the Ministry of Health, Labour and Welfare of Japan, were dispatched to the disaster area as a mobile medical team. A previous study with clean-up workers who participated in salvage activities after the Chernobyl nuclear accident revealed that the prevalence of depression, posttraumatic stress disorder (PTSD), and suicidal ideation 18years after the accident was higher than geographically matched controls and that radiation exposure level was associated with somatic and PTSD symptom severity [1]. However, there is a paucity of evidence available on the impact of subjective concerns over radiation exposure on health. The present study aimed to investigate early psychological distress among DMAT members who provided care to victims of the earthquake in the potentially dangerous situation of radiation exposure, and to ascertain whether an association exists between subjective concern over radiation exposure and psychological distress.

## Methods

### Participants

Participants were recruited from among the DMAT members deployed to the disaster area between March 11 and 22, 2011 following the earthquake. DMAT members are physicians, nurses, and operational coordination staff (medical or clerical staff who are neither physicians nor nurses) who are dispatched as a specially trained mobile medical team to conduct emergency medical relief activities during the acute phase of a large-scale disaster and in the event there are large numbers of injured or sick casualties. The inclusion criteria were 1) aged 18years or older, 2) a native Japanese speaker or non-native speaker with Japanese conversational abilities, and 3) physically and psychologically capable of understanding and providing consent for study participation.

### Enrollment procedures

This study was conducted as part of the clinical trial Attenuating posttraumatic distress with omega-3 polyunsaturated fatty acids among disaster medical assistance team members after the Great East Japan Earthquake. The detailed study procedures have been reported elsewhere [2]. Briefly, a written guide to the study was posted by the DMAT Office on the Emergency Medical Information System website, and affiliated hospitals with DMAT members were notified of the posting by their local municipalities. Survey packets were then mailed on April 2 to all DMAT members who had been deployed to the disaster area. Those willing to participate signed and returned the enclosed consent form by mail or fax to the DMAT Office by the closing date of April 12.

### Measures

Participants were surveyed using a self-report questionnaire that we prepared and operationalized the question in a binary (yes/no) way for this study. They provided information on the following items, in addition to those variables identified as risk factors for PTSD in previous research [3,4]: past history of psychiatric illness, period of deployment, stress prior to deployment, injury during deployment, experience of saving a child during deployment, experience of contact with corpses, concern over radiation exposure, and duration of time spent watching earthquake news reports. To evaluate past history of psychiatric illness, participants answered the question, Have you ever received any treatment for your mental health problem?. To evaluate stress prior to deployment, participants answered the question, Did you feel stressed just prior to deployment?. To evaluate concern over radiation exposure, participants answered the question, Were you concerned over radiation exposure during the deployment?. In regard to alcohol drinking, we prepared the following question Do you drink alcohol? and the answers I have never drank, I currently refrain from drinking and I drink. We defined current drinker as those respondents that chose the third answer I drink.

To evaluate psychological distress, respondents completed the Kessler 6 (K6) scale [5,6], the Center for Epidemiologic Studies Depression Scale (CES-D) [7,8], the Peritraumatic Distress Inventory (PDI) [9,10], and the Impact of Event Scale-Revised (IES-R) [11,12]. The K6 was designated to screen psychiatric disorders and mood and anxiety disorders. An adequate cutoff score on the K6 for serious mental illness with a score of less than 60 in the global assessment of functioning is 012 vs. 13 or more [13]. The cutoff score on the CES-D for depression is considered to be 16 or more [7]. The PDI assesses quantification of fear and sense of helplessness in the period during and immediately after a traumatic experience, and the cutoff score on the PDI for subsequent PTSD is 23 or more [14]. The IES-R evaluates PTSD symptoms and is the most widely used measure internationally in all forms of disaster-area research. Reliability and validity for each of these scales has been confirmed in the Japanese population [6,8,10,12]. Cronbachs alpha for the K6, CES-D, PDI, and IES-R in this sample was .89, .85, .83, and .94, respectively.

### Ethical considerations

This study protected the rights and welfare of the participants in the spirit of ethical guidelines outlined in the Declaration of Helsinki, and further respected the ethical principles of the Ministry of Health, Labour, and Welfare of Japan. The research plan (No. 201032) was deliberated upon and approved by the Ethics Committee of the National Disaster Medical Center on April 1, 2011. After providing a complete description of the study, written informed consent was obtained from all participants involved in the present study.

### Statistical analysis

Participants were assigned to 1 of 2 groups according to the presence or absence of concern over radiation exposure. Inter-group comparisons of the dependent variables, including K6, CES-D, PDI, and IES-R scores, were performed using an analysis of variance (ANOVA) after stratifying for sex. An analysis of covariance (ANCOVA) was then performed to examine the specific effect of concern over radiation exposure with age, occupation, disaster operation experience, duration of time spent watching earthquake news, and past history of psychiatric illness as covariates. Statistical significance was set at p<0.05 and all statistical analysis was performed using SPSS version 17 (SPSS Inc., Tokyo, Japan).

## Results

Of the 1,816 DMAT members deployed to the disaster area, 426 agreed to participate in the present study (24% response rate), of whom 424 (65.8% men, 34.2% women; mean age 38.2 (SD=7.5) years) completed a self-report questionnaire to assess psychological distress. Demographic characteristics are shown in Table 1. Physicians accounted for 24.1% of the sample, nurses 45.8%, and operational coordination staff 30.2%. Most of the women (89%) were nurses, while the occupational distribution was more dispersed for men; 33.7% were physicians, 23.3% were nurses, and 43.0% were operational coordination staff. Among all respondents, 9.2% were concerned over radiation exposure. The prevalence of probable psychological distress as determined by the K6 and CES-D was 4.0% and 21.4%, respectively. Both men and women with such concern showed significantly higher K6, CES-D, PDI, and IES-R scores than in those without concern (Table 2). After controlling for age, occupation, disaster operation experience, duration of time spent watching earthquake news, and past history of psychiatric illness, these associations remained significant in men, but did not remain significant in women for the CES-D and PDI scores. In addition, after excluding those with past history of psychiatric illness, ANOVA results showed no apparent change in the association between concern over radiation and psychological distress in men. In women, however, the association remained significant for the K6 (p=.029) but not for the CES-D, PDI, or IES-R (p=.097, p=.064, p=.064). ANCOVA results for women did not change the association. Moreover, for the ANCOVA, the addition of the two covariates experience of saving a child during deployment and experience of contact with corpses to the primary analysis of covariance did not change the association in men or women (data not shown).

**Table 1:** Demographic characteristics of participating DMAT members (n=424)

Variables	n (%)	mean (SD)	median (range)
Age (years)		38.2 (7.5)	
Period of deployment (days)			4 (121)
Sex, women	145 (34.2)		
Marital status, married	274 (64.6)		
Presence of a child (or children)	234 (55.2)		
Highest Education, university or higher	200 (47.2)		
Current smoker	100 (23.6)		
Current drinker	321 (75.7)		
Occupation			
Physician	102 (24.1)		
Nurse	194 (45.8)		
Operational coordination staff	128 (30.2)		
Past history of psychiatric illness, yes	8 (1.9)		
Stress prior to deployment, yes	104 (24.5)		
Injury during deployment, yes	2 (0.5)		
Disaster operation experience, yes	84 (19.8)		
Experience of contact with corpses, yes	47 (11.1)		
Experience of saving a child during deployment, yes	21 (5.0)		
Concern over radiation, yes	39 (9.2)		
Duration of time spent watching earthquake news			
<1 hour	107 (25.2)		
14 hours	292 (68.9)		
4 hours	25 (5.9)		

*DMAT*, disaster medical assistant team, *SD*, standard deviation

**Table 2:** Association between concern over radiation exposure and psychological distress among participating DMAT members

								
	concerned	not concerned		statistics	concerned	not concerned		statistics
	(n =23)	(n=256)			(n=16)	(n=129)		
	MeanSD	MeanSD	p value	p value*	MeanSD	MeanSD	p value	p value*
K6 total score	7.25.8	2.53.4	<.001	<.001	7.05.0	4.14.1	.011	.023
CES-D total score	16.39.7	10.26.8	<.001	<.001	18.410.8	13.18.7	.025	.084
PDI total score	20.08.7	11.46.5	<.001	<.001	18.79.1	14.47.2	.032	.072
IES-R total score	22.319.3	8.59.7	<.001	<.001	19.114.1	11.611.8	.021	.026

*DMAT* disaster medical assistant team, *SD* standard deviation, *K6* Kessler 6 scale, *CES-D* Center for Epidemiologic Studies Depression Scale, *PDI* Peritraumatic Distress Inventory, *IES-R* Impact of Event Scale-Revised

*controlled for age, occupation, disaster operation experience, duration of time spent watching earthquake news, and past history of psychiatric illness

Cronbachs alpha: for K6=.89, for CES-D=.85, for PDI=.83, for IES-R=.94

## Discussion

The present findings suggest that concern over radiation exposure was strongly associated with depressive and posttraumatic stress symptoms. Even among medical workers, who would likely be more knowledgeable than other public service workers about radiation exposure dangers, uncertainty about radiation exposure did have a significant adverse effect on their mental health. Thus, reliable and accurate information on radiation would be helpful for rescue workers, as well as residents, in disaster areas in order to minimize unnecessary psychological distress.

The prevalence of probable severe mental illness as determined by the K6 and depression as determined by the CES-D was 4.0% and 21.4%, respectively, in this study. For reference, the prevalence of depressive disorder was 14.9% among clean-up workers 18years after the Chernobyl accident [1]. An epidemiological study of a representative sample of community-dwelling Japanese (n=43,716) using the K6 showed that 6.7% had severe mental illness [15] and a large cohort study among Japanese workers with high and low socioeconomic status showed that 2027% of men and 3032% of women met the criteria for depression as determined by the CES-D [16]. Thus, the prevalence of depression in the present study was assumed to be equivalent to that of the general Japanese population or average workforce.

The present study has some limitations. First, 1,390 DMAT members who were invited to participate in the study did not respond, which could limit the external validity of the findings. The lack of response could be attributable to the fact that many rescue workers dedicated themselves to continuing important work at their own hospitals immediately after returning from their deployment and could not find the time to participate in this study. It remains unclear whether similar findings would have been obtained if the full cohort had been recruited successfully. There is the possibility that subjects who responded were more anxious or were more likely to be deployed in areas with high radiation exposure; however, this cannot be confirmed as it is not known if differences in demographic characteristics exist between DMAT members who responded and those who did not. Second, we could not directly examine whether individual personnel were actually exposed to radiation or examine the geographic sites where they were deployed. We also did not assess the exact nature of the disaster work they undertook during their deployment, and this constitutes a weakness in the assessment of the traumatic event. Third, it is important to note that over 70% of participants in the present study were nurses and physicians, which may limit the generalizability of our findings to other rescue worker populations such as the police, local fire departments, and self-defense force, although they all have similar occupational responsibility to the public. Fourth, concern over radiation exposure might be influenced by the level of psychological distress. A longitudinal study is needed to examine the causality. Further research is needed to examine more closely the association between actual exposure level of radiation and psychological distress in individuals at risk from radiation exposure.

## Conclusion

In conclusion, the results suggested that concern over radiation exposure was strongly associated with psychological distress among rescue workers.

## Competing interests

The authors declare that they have no competing interests in relation to this manuscript.

## Authors contributions

YM and DN conceived the study and drafted the original protocol. YM, DN, NN, TS, KH, TH, and YK participated in refining the protocol. YK managed the enrolment procedure and overall control of the study. YM, DN, and HN recruited and contacted the participants. All authors contributed to the design of the study, and TS and NN calculated sample size and decided the analytic strategy. All authors read and approved the final manuscript.

## Pre-publication history

The pre-publication history for this paper can be accessed here:

http://www.biomedcentral.com/1471-2458/12/249/prepub

## References

[B1] Loganovsky K Havenaar JM Tintle NL Guey LT Kotov R Bromet EJ The mental health of clean-up workers 18years after the Chernobyl accident Psychol Med 2008 38 04 481 488 18047772 10.1017/S0033291707002371

[B2] Matsuoka Y Nishi D Nakaya N Sone T Hamazaki K Hamazaki T Koido Y Attenuating posttraumatic distress with omega-3 polyunsaturated fatty acids among disaster medical assistance team members after the Great East Japan Earth quake: The APOP randomized controlled trial BMC Psychiatry 2011 11 132 21846343 10.1186/1471-244X-11-132 PMC3167761

[B3] Epstein RS Fullerton CS Ursano RJ Posttraumatic Stress Disorder Following an Air Disaster: A Prospective Study Am J Psychiatry 1998 155 7 934 938 9659860 10.1176/ajp.155.7.934

[B4] Schlenger WE Caddell JM Ebert L Jordan BK Rourke KM Wilson D Thalji L Dennis JM Fairbank JA Kulka RA Psychological reactions to terrorist attacks: findings from the National Study of Americans' Reactions to September 11 JAMA 2002 288 5 581 588 12150669 10.1001/jama.288.5.581

[B5] Kessler RC Andrews G Colpe LJ Hiripi E Mroczek DK Normand SL Walters EE Zaslavsky AM Short screening scales to monitor population prevalences and trends in non-specific psychological distress Psychol Med 2002 32 6 959 976 12214795 10.1017/s0033291702006074

[B6] Furukawa TA Kessler RC Slade T Andrews G The performance of the K6 and K10 screening scales for psychological distress in the Australian National Survey of Mental Health and Well-Being Psychol Med 2003 33 2 357 362 12622315 10.1017/s0033291702006700

[B7] Radloff LS The CES-D scale: a self-report depression scale for research in the general population Appl Psychol Meas 1977 1 385 401

[B8] Shima S Shikano T Kitamura T A new self-report depression scale (in Japanese) Seishinigaku 1985 27 717 723

[B9] Brunet A Weiss DS Metzler TJ Best SR Neylan TC Rogers C Fagan J Marmar CR The Peritraumatic Distress Inventory: a proposed measure of PTSD criterion A2 Am J Psychiatry 2001 158 9 1480 1485 11532735 10.1176/appi.ajp.158.9.1480

[B10] Nishi D Matsuoka Y Noguchi H Sakuma K Yonemoto N Yanagita T Homma M Kanba S Kim Y Reliability and validity of the Japanese version of the Peritraumatic Distress Inventory Gen Hosp Psychiatry 2009 31 1 75 79 19134513 10.1016/j.genhosppsych.2008.09.002

[B11] Weiss DS Marmar CR Wilson JP, Keane TM The Impact of Event Scale Revised Assessing psychological trauma and PTSD 1997 The Guilford Press, New York 399 411

[B12] Asukai N Kato H Kawamura N Kim Y Yamamoto K Kishimoto J Miyake Y Nishizono-Maher A Reliability and validity of the Japanese-language version of the impact of event scale-revised (IES-R-J): four studies of different traumatic events J Nerv Ment Dis 2002 190 3 175 182 11923652 10.1097/00005053-200203000-00006

[B13] Kessler RC Barker PR Colpe LJ Epstein JF Gfroerer JC Hiripi E Howes MJ Normand SL Manderscheid RW Walters EE Screening for serious mental illness in the general population Arch Gen Psychiatry 2003 60 2 184 189 12578436 10.1001/archpsyc.60.2.184

[B14] Nishi D Matsuoka Y Yonemoto N Noguchi H Kim Y Kanba S Peritraumatic Distress Inventory as a predictor of post-traumatic stress disorder after a severe motor vehicle accident Psychiatry Clin Neurosci 2010 64 2 149 156 20447011 10.1111/j.1440-1819.2010.02065.x

[B15] Kuriyama S Nakaya N Ohmori-Matsuda K Shimazu T Kikuchi N Kakizaki M Sone T Sato F Nagai M Sugawara Y Factors associated with psychological distress in a community-dwelling Japanese population: the Ohsaki Cohort 2006 Study J Epidemiol 2009 19 6 294 302 19749498 10.2188/jea.JE20080076 PMC3924098

[B16] Inoue A Kawakami N Interpersonal conflict and depression among Japanese workers with high or low socioeconomic status: findings from the Japan Work Stress and Health Cohort Study Soc Sci Med 2010 71 1 173 180 20444532 10.1016/j.socscimed.2010.02.047

